# Short-time response of soil ecological stoichiometry on aboveground biomass under fertilizer application of mixed grass pasture in the Northern Tibetan Plateau

**DOI:** 10.1371/journal.pone.0326265

**Published:** 2025-07-21

**Authors:** Juanjuan Zhou, Fei Xia, Wenxia Cao, Wei Wei

**Affiliations:** 1 Key Laboratory of Grassland Ecosystem, Ministry of Education, College of Pratacultural Science, Gansu Agricultural University, Lanzhou, China; 2 Institute of Pratacultural Science, Tibet Academy of Agricultural and Animal Husbandry Science, Lhasa, China; 3 State Key Laboratory of Hulless Barley and Yak Germplasm Resources and Genetic Improvement, Lhasa, China; University of Minnesota, UNITED STATES OF AMERICA

## Abstract

The construction of artificial grasslands using native species is an effective measure to restore degraded grassland. In this study, three native grass species, *Elymus nutans*, *Elymus tangutorum* and *Poa albertii* subsp. poophagorum, domesticated in the northern Tibetan Plateau were used as test subjects. Three monocultures of *E. nutans, E. tangutorum* and *P. albertii* subsp. poophagorum and four mixed combinations *E. nutans +E. tangutorum*, *E. nutans +P. albertii* subsp. poophagorum, *E. tangutorum +P. albertii* subsp. poophagorum and *E. nutans + E. tangutorum + P. albertii* subsp. poophagorum were set up, with 7 sowing types as the main zones, and nested fertilization and non-fertilization as the secondary zones. The optimal sowing combinations were selected to clarify the community growth dynamics of different grass pasture, and to investigate the transgressive overyielding and diversity effects of artificial grassland and the response of soil chemometrics to fertilizer application, with a view to providing a scientific basis for ecological restoration of degraded grassland in the northern Tibetan Plateau. The results show that the different sowing combinations of grassland all exhibit obvious dynamic population change in the growing season, with aboveground biomass peaking on 20 September. The type of *E. nutans* is the dominant community of the mixed combinations. The forage yield was highest in the combination, and the fertilization treatment significantly increased the forage yield. *E. nutans + E. tangutorum + P. albertii* subsp. poophagorum mixed sowing with fertilizer application is the recommended methodology to establish artificial grassland in the northern Tibetan Plateau. The relative yield totals of the mixed combinations were all greater than 1, and all were transgressive overyielding. Combined with the analysis of the distribution of soil ecological stoichiometric characteristics under fertilizer and non-fertilizer treatments, it was found that aboveground biomass and transgressive overyielding coefficients responded to soil ecological stoichiometry in completely different ways. Under the unfertilized treatment, soil C, N and P are regulated primarily through microbial stoichiometry under the resource dependence of soil dissolved nutrient stoichiometry, which affected aboveground of artificial grassland. Under the fertilizer treatment, microbially mediated extracellular enzyme stoichiometry was dynamically changing to regulate the new substrate environmental supply and demand balance derived from fertilizer application. Future studies should examine the long-term impacts of these combinations across diverse environments by integrating complementary restoration techniques to improve artificial grassland sustainability, thereby offering scientific support for regional ecological restoration efforts.

## 1 Introduction

The landscape of grassland is one of Chinese major natural landscapes, which play an important role in the C and N cycles of terrestrial ecosystems [1]. Tibetan Plateau is located on the ‘roof of the world’ as the ecological barrier in the southwest of China, with high cold and oxygen deprivation. Natural grassland is the largest and most important ecosystem in Tibet [2]. The alpine grassland of the Tibetan Plateau covers an area of 1.27 × 10^8^ km^2^, accounting for 50.90% of the total area [3]. For a long time, the natural grassland ecosystem of Tibet has not only provided the material basis for the production and livelihood of local farmers and herdsmen, but is also an important ecological natural green barrier and a major source of water resources in China [4]. And its ecological environment directly or indirectly affects the ecological security of the region and the surrounding areas, and has a great impact on the production and livelihood of local farmers and herdsmen [5]. In recent years, with the increasing population and demand for livestock products, Tibetan grassland have been affected by a combination of factors such as overgrazing, indiscriminate logging, rodent pest and mining damage, resulting in serious damage to the region’s grassland ecosystem. The area of degraded grassland is about 4.50 × 10^7^ km^2^ and nearly 1/3 of the alpine grassland is in a serious state of degradation [6], even forming a large area of secondary bare land that cannot be restored naturally. But the restoration of degraded grasslands is a long process, taking tens or even hundreds of years [7]. These problems have led to poor improvements in grassland livestock farming. As the main source of forage for livestock in grassland areas, hay harvested from natural pastures can effectively solve the problem of grass-livestock unbalanced and plays a key role in ensuring that livestock can safely survive the winter [8,9]. Long-term haying can lead to soil nutrient depletion, which will eventually be reflected in the productivity of the grass, thus affecting the yield of the forage [10].

Currently, establishing stable, high-quality, high-yield artificial grasslands is an effective approach to restoring severely degraded grasslands and secondary bare grounds [4,11]. This strategy not only alleviates grazing pressure on natural grasslands but also enhances grassland productivity and restores ecological functions [7,12–14]. Techniques such as fertilization, mixed sowing, root cutting, and fencing restoration play crucial roles in this effort. Multi-species communities can effectively increase environmental resource use efficiency [15,16], resulting in higher productivity and stability over time compared to single-species or monoculture systems [17–19].

A key aspect of grassland restoration involves the concept of transgressive overyielding, which refers to the phenomenon where multi-species communities outperform the combined yield of individual species when grown in monoculture. This effect is particularly important in artificial grasslands, as it suggests that species interactions in mixed plantings can enhance overall productivity beyond what would be expected from the sum of individual species’ performances. Understanding the mechanisms behind transgressive overyielding is crucial for optimizing restoration practices, as it can guide the selection of plant species that maximize yield and ecological benefits.

Ecological stoichiometry, an emerging interdisciplinary field, integrates ecology and stoichiometry principles to study the mass balance of key chemical elements—primarily carbon, nitrogen, and phosphorus—in ecological interactions [20,21]. It studies the mass balance of multiple chemical elements, primarily carbon, nitrogen, and phosphorus, in ecological interactions [22], gradually becoming a critical tool in ecological research [21]. In soil, carbon concentration depends on inputs from plant biomass, animal matter, necromass, and humus, as well as their humification processes [23]. Nitrogen derives mainly from litter decomposition, biological nitrogen fixation, and dissolved nitrogen from precipitation [24]. Soil nitrogen and phosphorus are essential chemical elements for plant growth and crucial factors in terrestrial ecosystems for determining species abundance [25]. Soil nutrient supply, plant nutrient demand, and restitution maintain dynamic elemental ratios [26]. Numerous studies indicate that ecological stoichiometry serves as an indicator of microbial metabolic limitations [27]. Stoichiometric ratios of soil carbon, nitrogen, and phosphorus reflect microbial metabolic constraints in degraded grasslands. Soil microbial biomass, as a nutrient source and reservoir, reflects grassland soil nutrient status [28]. These ratios can be utilized to assess microbial metabolic limitations and reflect the nutrient needs of the grassland ecosystem [29]. Additionally, extracellular enzymes play a vital catalytic role in organic matter decomposition and nutrient cycling [30]. Exploring the stoichiometry of extracellular enzymes involved in carbon, nitrogen, and phosphorus cycling can reflect the biogeochemical balance between microbial metabolism, nutrient demand, and environmental nutrient effectiveness [31–33]. Ecological stoichiometry provides insights into how fertilization, mixed sowing, and related restoration techniques influence plant competitive strategies. Ecological stoichiometry also informs how restoration techniques, such as fertilization and mixed sowing, influence plant competitive strategies. Fertilization often shifts plant reproductive allocation, favoring taller species that are efficient at nutrient uptake, while smaller species may be excluded from the community [34–35]. Simultaneously, mixed sowing and fertilization have complementary effects, alleviating nitrogen limitations, increasing forage yield, significantly improving soil nutrients, and maintaining grassland stability [36–37].

In degraded grassland ecosystem, soil degradation lags behind vegetation degradation and is a more serious degradation than vegetation degradation. Therefore, the recovery time for soil degradation is also much longer than that for vegetation. Appropriate fertilizer application is an important measure to ensure the balance between substance input and output and to achieve sustainable development [38]. Constrained by the cold and arid climatic conditions of the northern Tibetan Plateau, the adaptation of grass species is the main issue facing the construction of artificial grassland for the restoration of alpine degraded grassland in the northern Tibetan Plateau. The selection of native species for grassland restoration that close to the zonal vegetation of the degraded grassland restoration areas becomes crucial to ensure the stability and sustainability of the re-established grassland communities.

The present study investigates the dynamics of forage yield under different sowing combinations and fertilization conditions, focusing on three native grasses in artificial grassland communities in the northern Tibetan Plateau. This study aims to clarify the causes of the transgressive overyielding effect and its correlation with soil stoichiometry. Additionally, it seeks to explore how different native species adapt to the environment, their coexistence and competition within community compositions, and the relationship between aboveground biomass and soil ecological stoichiometry. The findings of this research will provide valuable insights into the ecological restoration of degraded grasslands in the northern Tibetan Plateau.

## 2 Materials and methods

### 2.1 Study area

This study was carried out in the city of Nagqu, Tibet Autonomous Region, in the heart of the Tibetan Plateau (92°07′ E, 31°26′ N). Study area is characterized by a dry, windy, semi-arid monsoon climate, with a combination of rain and heat. The annual average temperature is −2.9 °C, the mean annual precipitation is 400 mm, mainly occurring between June and September. There is no absolute frost-free period. The study area is a flat, heavily degraded alpine grassland with *Koeleria argentea*, *Potentilla bifurca*, *Stipa purpurea*, *Stracheya tibetifca*, *Heteropappus hispidu* and *Lepidium apetalum* as the main miscellaneous grasses.

### 2.2 Methods

In this study, the native grass species were *E. nutans*, *E. tangutorum* and *P. albertii* subsp. poophagorum, all harvested from the northern Tibetan Plateau. They were cultivated and domesticated for nine years at the ‘Northern Tibetan Alpine Grassland Ecological and Technological Park’ in Nagqu City (31°26′ N, 92°01′ E, 4512 m) then used to establish a perennial mixed artificial grassland.

The experiment was sown in early June 2019 as monocultures and mixed combinations. The monocultures were done as a single sowing of *E. nutans* (Ⅰ.), *E. tangutorum* (Ⅱ.) and *P. albertii* subsp. poophagorum (Ⅲ.) at 2.25 g·m^-2^, 2.25 g·m^-2^ and 1.50 g·m^-2^, respectively. The mixed sowings were two mixes and three mixes with four sowing combinations, *E. nutans* *+ E. tangutorum* (Ⅰ. + Ⅱ.), *E. nutans + P. litwinowiana* (Ⅰ. + Ⅲ.), *E. tangutorum + P. albertii* subsp. poophagorum (Ⅱ. + Ⅲ.) and *E. nutans + E. tangutorum + P. albertii* subsp. poophagorum (Ⅰ. + Ⅱ. + Ⅲ.) (Table 1). The amount of individual sowing rate of each forage type in two mixes was 50.00%, 33.30% of the monocultures sowing rate for the three mixtures, 7 pasture types in total. The sowing pasture was random-distributed and replicated 4 times, each time on 3 m x 4 m plots with a 1 m buffer strip between the sowing combinations. Each sowing pasture was divided into two sub-zones of 3.0 m x 2.0 m which containing two treatments, one is control (No-Fert.) and the other is fertilization (Fert.), with a 0.5 m horizontal and vertical separation strip. The fertilizer treatment was (NH_4_)_2_HPO_4_, applied once in early June of the second year after sowing (sprinkler irrigation after fertilization). The fertilizer application was 60.00 g·m^-2^ (10.80 g·m^-2^ for pure N and 27.60 g·m^-2^ for pure P). The sowing was sown at a depth of 3–5 cm, in rows 25 cm apart, mulched after sowing, and weeded twice by hand after emergence. The experimental site undergoes a natural winter, with pre-winter watering and no mulching measures.

**Table 1:** Experimental treatment.

Treatment	Pasture type	No.
Fert.	*El. N*	Ⅰ.
	*El. T*	Ⅱ.
	*Po. A*	Ⅲ.
	*El. N* + *El. T*	Ⅰ. + Ⅱ.
	*El. N* + *Po. A*	Ⅰ. + Ⅲ.
	*El. T* + *Po. A*	Ⅱ. + Ⅲ.
	*El. N *+ *El. T *+* Po. A*	Ⅰ. + Ⅱ. + Ⅲ.
No-Fert.	*El. N*	Ⅰ.
	*El. T*	Ⅱ.
	*Po. A*	Ⅲ.
	*El. N *+ *El. T*	Ⅰ. + Ⅱ.
	*El. N* + *Po. A*	Ⅰ. + Ⅲ.
	*El. T* + *Po. A*	Ⅱ. + Ⅲ.
	*El. N* + *El. T* + *Po. A*	Ⅰ. + Ⅱ. + Ⅲ.

Sampling was carried out on the 20th of each month from July to September (plant growing season) after sowing. To eliminate interference between adjacent sowing treatments, one row on each side of treatments was left unmeasured and unsampled. To ensure that the biomass collection from July to September was within the same sample site, two randomly fixed 0.25 m x 0.25 m squares were design before the first fertilizer application. The aboveground biomass was harvested, dried at 65°C to a constant weight. Soil samples was collected at the last harvest. In each quadrat, a homogenized sample (0–10 cm) was collected from the four corners and center using a soil auger (20-cm depth and 5-cm diameter). Soil samples were sieved through a 2-mm mesh to remove large stones and roots. Each soil sample was divided into two parts, with one of those subsamples air-dried and another part of subsamples retained at 4°C to measure the soil properties and extracellular enzymes.

### 2.3 Measurement methods

Soil properties: the following parameters were measured: soil organic carbon (SOC, g·kg^-1^), soil total nitrogen (TN, g·kg^-1^), soil total phosphorus (TP, g·kg^-1^), soil dissolved organic carbon, nitrogen and inorganic phosphorus (DC, DN and DP; mg·kg^-1^) and soil microbial biomass carbon, nitrogen and phosphorus (MBC, MBN, and MBP; mg·kg^-1^). SOC was measured by potassium dichromate oxidation [39]. TN and TP were determined by standard protocols [40]. The concentrations of MBC, MBN and MBP were measured using the chloroform fumigation-extraction method according to Vance et al., (1987) [41] and Brookes et al., (1985) [42], respectively. Briefly, one part was fumigated with CHCl_3_ for 24 h at 25°C, and the others were non-fumigated. The fumigated and non-fumigated soil were extracted in 50 ml 0.5 M K_2_SO_4_ at a ratio of 1 : 4 (W/V) for MBC and MBN, and 50 ml 0.5 M NaHCO_3_ at a ratio of 1 : 20 (W/V) for MBP. MBC, MBN and MBP were calculated according to the difference between fumigated and non-fumigated values and adjusted using conversion coefficients E, where EC, EN and EP were 0.45, 0.45 and 0.40, respectively [43]. The concentrations of DC, DN and DP were calculated from the non-fumigated values [44].

Soil extracellular enzymes: the following extracellular enzyme activities were measured, which are associated with the microbial acquisition of C (BG, β-1,4-glucosidase; CBH, β-D-cellobiohydrolase), N (NAG, β-N-acetyl glucosaminidase; LAP, Leucine aminopeptidase), and P (AP, Alkaline phosphatase). 1 g fresh soil was added to 250 mL of 0.5 M acetate buffer and dispersed by ultrasonic disaggregation (50 J/s for 120s) [45]. Using 96-well plates standard fluorimetric techniques for analysis (S1 Table) [46,47].

### 2.4 Statistical analysis

Statistical distributions of aboveground biomass, relative total yield (RTY), over yield (OY), transgressive overyielding effect (OY_1_ and OY_2_), total nutrient stoichiometry (TNS) dissolved nutrient stoichiometry (DNS), microbial biomass stoichiometry (MBS) and, extracellular enzyme stoichiometry (EES) were assessed through expected probability (Q-Q) plots, with skewness and kurtosis quantified for all indicators. All data were tested for homogeneity of variance before analysis, and those with variance were log-transformed. The statistical analyses were carried out with R software v3.4.2 (http://www.r-project.org). We used the Redundancy analysis (RDA) and varpart functions of the ‘vegan’ package to run redundancy analysis (RDA) and variance partitioning analysis (VPA), respectively. VPA was used to analysis the explanation of the impact of TNS, DNS, MBS, EES on yield indicators. RDA was to explore the relationship between stoichiometric characteristics and yield indicators.

### 2.5 The competitiveness of inter-species

The relative yield total (RYT) can characteristic the competitiveness between mixed species. It is calculated as follows:


$$RYT=\frac{Y_{ij}}{Y_{ii}}+\frac{Y_{ji}}{Y_{jj}}\mathrm{\backslash ]}$$

where, Y_ij_ is the biomass of i specie in mixed sowing. Y_ji_ is the biomass of j specie in mixed sowing. Y_jj_ is the biomass of i species in monoculture. Where RYT >  1, the interspecific competition of the mixing sowing is less than the intraspecific competition, showing a symbiotic relationship. When RYT = 1, the interspecific competition of the mixing sowing is equal to the intraspecific competition. When RYT < 1, the interspecific competition of the mixing sowing is greater than the intraspecific competition, showing an antagonistic relationship [48].

### 2.6 The effect of transgressive overyielding

Over-yield (OY) is the difference between the mixed aboveground biomass and the mean monoculture aboveground biomass of each species in the community.


$$OY=B_{mc}-B_{s}\mathrm{\backslash ]}$$

where, B_mc_ is the above-ground biomass of the mixed community. Bs is the average above-ground biomass of each species in the mixed community. When OY >  0 indicating over-production.

Transgressive overyielding effect 1 (OY_1_) is the above-ground biomass of the mixed community exceeds the above-ground biomass of the monoculture species with the highest biomass in that community. It emphasises the differences between the mixed species and the link with over-production effect [49].


$${OY}_{1}=B_{mc}-maxB_{imno}\mathrm{\backslash ]}$$

where, max B_imno_ is the above-ground biomass of the highest productivity species in the mixed community. When OY_1_ >  0, indicating an transgressive overyielding effect.

Transgressive overyielding effect 2 (OY_2_) is the above-ground biomass of a mixed community exceeds the average above-ground biomass of the monoculture species within that community. It explains the relationship between the above-ground biomass of the mixed community and monoculture species of the mixed combination [49].


$${OY}_{2}=\frac{B_{mc}-B_{imno}}{B_{imno}}\mathrm{\backslash ]}$$

where, B_imno_ is the mean monocultures above-ground biomass of each species in the mixed community. When OY_2_ >  0, indicating an transgressive overyielding effect.

## 3 Result

### 3.1 Dynamic change of aboveground biomass indicators under different mixed combination

#### Aboveground biomass.

As the phenological period progressed, the aboveground biomass increased gradually in both the monoculture and mixed treatments, reaching a peak on 20 September (Table 2). In the monoculture treatments, rapid growth occurred from 20 July to 20 August. In particular, the forage yield of treatments Ⅰ. was higher than treatments Ⅱ., and the yield of treatments Ⅲ. was the lowest. The application of fertilize significantly increased the forage yield in the early period. The total forage yield was highest in treatment Ⅰ. + Ⅱ. + Ⅲ., followed by Ⅰ. + Ⅱ. and lowest in treatment Ⅰ. + Ⅲ. The forage yield increased more than 88.23% after fertilization. In the mixed sowing treatments, *E. nutans* was overwhelmingly dominant except on 20 August among the mixed combination of Ⅰ. + Ⅱ., Ⅰ. + Ⅲ. And Ⅰ. + Ⅱ. + Ⅲ. It’s forage yield accounted for more than 70% of total yield at that time. On 20 August, the aboveground biomass of *E. nutans* in the mixed combination of Ⅰ. + Ⅱ. + Ⅲ. decreased to 45.68% and 48.39% in No-Fert. and Fert. treatments, respectively. The total percentage of *E. tangutorum* and *P. albertii* subsp. poophagorum was over than 50%. The contribution of *P. albertii* subsp. poophagorum to the forage yield was greater in mixed combination of Ⅰ. + Ⅲ. than Ⅱ. + Ⅲ.

**Table 2:** Dynamic change of aboveground biomass under different grass pasture (g·m^-2^).

No.	Component	Sampling time (Month-day)					
		Fert.			No-Fert.		
		7-20	8-20	9-20	7-20	8-20	9-20
Ⅰ., Ⅱ., Ⅲ.	Ⅰ. -- *El. N*	361.51	1003.47	1972.35	91.49	620.59	91.49
	Ⅱ. -- *El. T*	334.29	702.02	801.46	32.14	337.65	39.64
	Ⅲ. -- *Po. A*	208.58	1177.15	2188.39	36.16	634.82	36.16
Ⅰ. + Ⅱ.	Ⅰ. -- *El. N*	378.50	775.79	1848.49	54.98	368.69	812.92
	Ⅱ. -- *El. T*	38.51	375.08	778.99	21.75	273.33	169.60
	Total	417.01	1150.87	2627.48	76.73	642.02	982.52
Ⅱ. + Ⅲ.	Ⅱ. -- *El. T*	354.55	841.55	1755.33	87.47	464.21	792.9
	Ⅲ. -- *Po. A*	107.82	348.57	336.59	24.97	278.80	128.28
	Total	462.37	1190.13	2091.91	112.43	743.02	921.07
Ⅰ. + Ⅲ.	Ⅰ. -- *El. N*	165.06	588.58	1074.38	53.15	365.41	314.18
	Ⅲ. -- *Po. A*	70.91	355.11	329.16	33.72	332.90	141.25
	Total	235.98	943.69	1403.54	86.87	698.31	455.43
Ⅰ. + Ⅱ. + Ⅲ.	Ⅰ. -- *El. N*	434.00	958.97	2280.90	128.17	489.80	1019.95
	Ⅱ. -- *El. T*	22.29	557.55	268.95	45.10	310.07	174.60
	Ⅲ. -- *Po. A*	19.06	480.08	243.19	36.73	267.79	168.54
	Total	475.35	1996.60	2793.04	209.99	1067.67	1363.09

### 3.2 The relative yield total (RYT) and Over-yield (OY)

**Fig 1:**
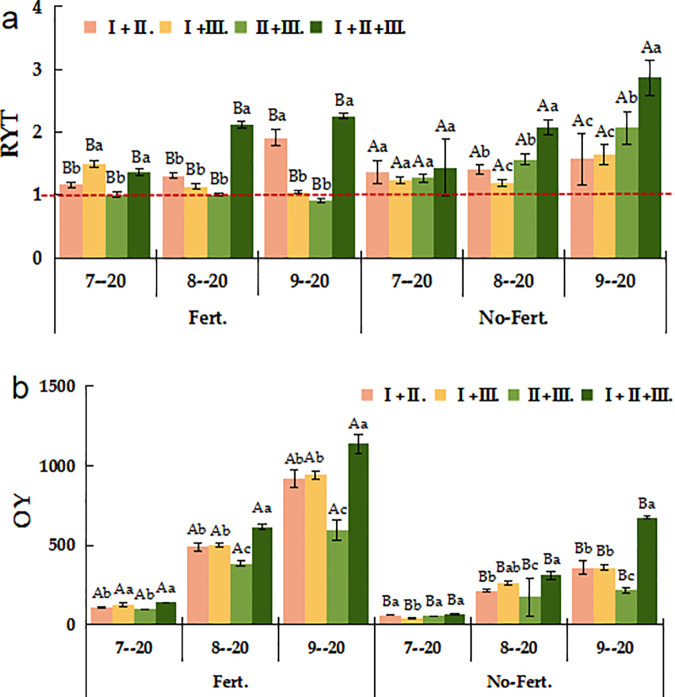
The RYT and OY of different sowing combinations under Fert. and No-Fert. treatments. Note: Uppercase letters mean different fertilization treatments of the same sowing combination (*P < 0.05*); Different lowercase letters mean different sowing types of the same fertilization treatments (*P < 0.05*). RYT, relative yield total; OY, Over-yield; Ⅰ. + Ⅱ., *El. N* + *El. T*; Ⅰ. + Ⅲ., *El. N* + *Po. A*; Ⅱ. + Ⅲ., *El. T* + *Po. A*; Ⅰ. + Ⅱ. + Ⅲ., *El. N* + *El. T* + *Po. A*. The same below.

As Fig 1a shown, the RYT values of mixed combinations Ⅰ. + Ⅱ., Ⅰ. + Ⅲ., Ⅱ. + Ⅲ. and Ⅰ. + Ⅱ. + Ⅲ. under No-fert. and Fert. treatments were greater than 1.0. The highest RYT values of 1. 58, 1. 65, 2. 07 and 2.87 were recorded on 20 September for the mixed combination of Ⅰ. + Ⅱ. + Ⅲ. This indicates that intra-species competition is less than inter-species competition for each species in the *E. nutans +E. tangutorum*, *E. nutans +P. albertii* subsp. poophagorum and *E. nutans + E. tangutorum + P. albertii* subsp. poophagorum mixed combination during different phenological periods. The RYT values for the mixed Ⅱ. + Ⅲ. were greater than 1. 0 under Fert. treatment and less than 1.0 under No-Fert. treatment. This indicates that intra-species competition was greater than inter-species competition in the *E. nutans +P. albertii* subsp. poophagorum mixed combination, and that inter-species competition increased after fertilization and tended to be closer to intra-species competition. All mixed combination except the Ⅱ. + Ⅲ. were over-productive compared to the monoculture treatment, the Ⅰ. + Ⅱ. + Ⅲ. mixed combinations’ overyielding were 139.39, 612.99 and 1135.917 g·m^-2^ in July, August and September under the Fert. treatment, respectively (Fig 1b).

### 3.3 Over-yield 1 (OY_1_) and Over-yield 2 (OY_2_)

**Fig 2:**
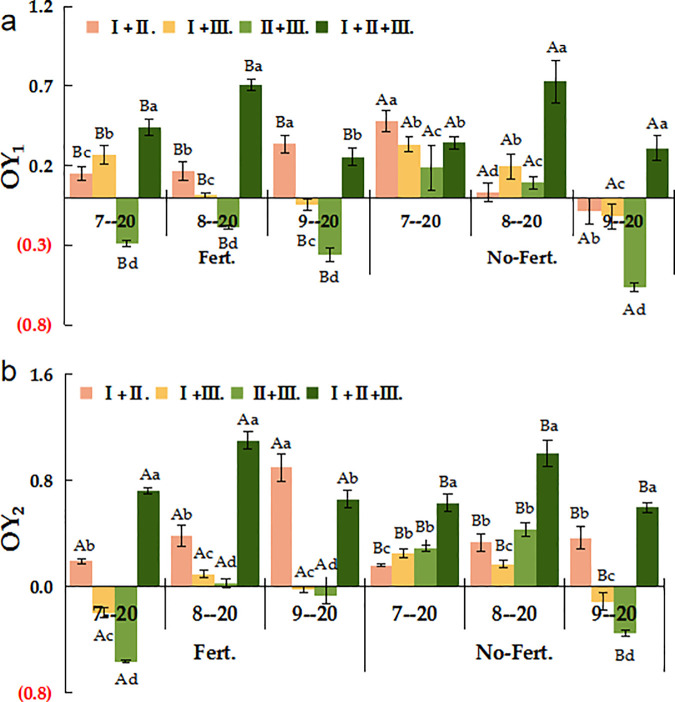
The OY_1_ and OY_2_ of different sowing combinations under Fert. and No-Fert. treatments. Note: Uppercase letters mean different fertilization treatments of the same sowing combination (*P < 0.05*); Different lowercase letters mean different sowing types of the same fertilization treatments (*P < 0.05*). OY_1_, Transgressive overyielding effect 1; OY_2_, Transgressive overyielding effect 2; Ⅰ. + Ⅱ., *El. N* + *El. T*; Ⅰ. + Ⅲ., *El. N* + *Po. A*; Ⅱ. + Ⅲ., *El. T* + *Po. A*; Ⅰ. + Ⅱ. + Ⅲ., *El. N* + *El. T* + *Po. A*. The same below.

**Fig 3:**
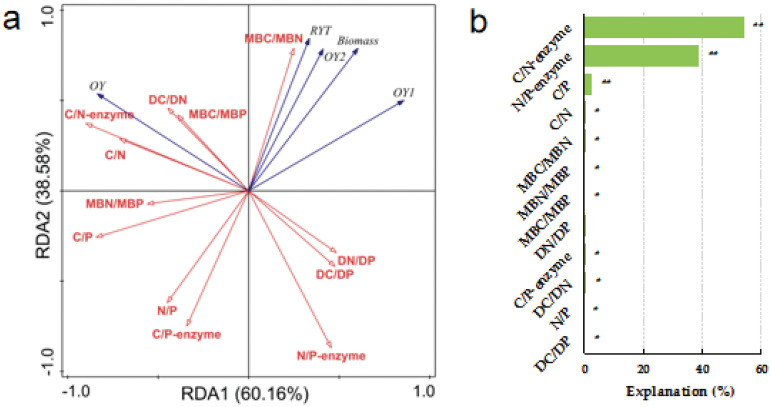
Redundancy analysis (RDA) identifies the relationships between aboveground biomass indicators and soil ecological stoichiometric ratios under Fert. treatment. Note: RYT, relative yield total; OY, Over-yield; OY_1_, Transgressive overyielding effect 1; OY_2_, Transgressive overyielding effect 2; Soil total nutrients stoichiometry (C/N, C/P and N/P); Soil dissolved nutrients stoichiometry (DC/DN, DC/DP and DN/DP); Soil microbial biomass stoichiometry (MBC/MBN, MBC/MBP and MBN/MBP) and soil extracellular enzyme stoichiometry (C/N-enzyme, C/P-enzyme and N/P-enzyme) under Fert. treatments. The significance level of the simple effect was P<0.05* and P<0.01**.

As Fig 3 shown, the Fert. treatment significantly increased the transgressive overyielding effect of the mixed combinations (*P < 0.05*). Under Fert. treatment, the value of OY_1_ was greater than 0 for all mixed combinations except Ⅱ. + Ⅲ. in different phenological stages which indicated the transgressive overyielding effect 1. Under No-Fert. treatment, the value of OY_1_ was greater than 0 only for Ⅰ. + Ⅱ. + Ⅲ. mixed combination on 20 September, while all other values were negative indicated the absence of transgressive overyielding effect 1 (Fig 2a). The value of OY_2_ was significantly higher under the Fert. treatment than the No-Fert. treatment for all mixed combinations in different phenological stages (*P < 0.05*). Under the Fert. treatment, there was a transgressive overyielding effect 2 in all mixed combinations except for the Ⅰ. + Ⅲ. and Ⅰ. + Ⅱ. + Ⅲ., where the value of OY_2_ was less than 0 in 20 July and September. Under the No-Fert. treatment, there was no transgressive overyielding effect 2, except in the Ⅰ. + Ⅲ. and Ⅰ. + Ⅱ. + Ⅲ. mixed combinations on 20 September (Fig 2b).

### 3.4 Influential factors of forage yield indicators

The factors influencing forage yield indicators between different sowing combinations under fertilization treatment were analyzed based on RDA (Fig 3). Aboveground biomass, RYT, OY_1_ and OY_2_ were significantly and positively correlated with MBC/MBN (*P < 0.01*), the correlation coefficients were 0.775, 0.704, 0.606 and 0.666, respectively. While significantly and negatively correlated with C/P, N/P and C/P-enzyme (*P < 0.01*). RYT also significantly and negatively correlated with N/P-enzyme (*P < 0.01*). OY significantly and positively correlated with C/N, C/P, DC/DN, C/N-enzyme and N/P-enzyme (*P < 0.01*), while significantly and negatively correlated with DC/DP and N/P-enzyme (*P < 0.01*). The ratios of C/N-enzyme, N/P-enzyme and C/P (54.0% and 38.9%) explained the most under Fert. treatment, respectively.

**Fig 4:**
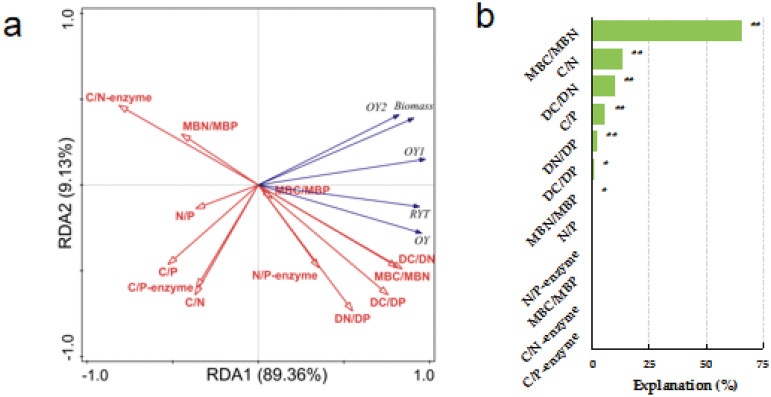
Redundancy analysis (RDA) identifies the relationships between aboveground biomass indicators and soil ecological stoichiometric ratios under No-Fert. treatment. Note: RYT, relative yield total; OY, Over-yield; OY_1_, Transgressive overyielding effect 1; OY_2_, Transgressive overyielding effect 2; Soil total nutrients stoichiometry (C/N, C/P and N/P); Soil dissolved nutrients stoichiometry (DC/DN, DC/DP and DN/DP); Soil microbial biomass stoichiometry (MBC/MBN, MBC/MBP and MBN/MBP) and soil extracellular enzyme stoichiometry (C/N-enzyme, C/P-enzyme and N/P-enzyme) under No-Fert. treatments. The significance level of the simple effect was P<0.05* and P < 0.01**.

The factors influencing forage yield indicators between different sowing types under No-Fert. treatment was analyzed based on RDA (Fig 4). Aboveground biomass and OY_2_ were significantly and positively correlated with DC/DN and MBC/MBN. While significantly and negatively correlated with C/N and C/N-enzyme (*P < 0.01*). Aboveground biomass also significantly and negatively correlated with C/P and C/P-enzyme. RYT, OY and OY_1_ were significantly and positively correlated with DC/DN, DC/DP, DN/DP and MBC/MBN. While significantly and negatively correlated with MBN/MBP and C/N-enzyme (*P < 0.01*). OY_1_ also significantly and negatively correlated with C/N and C/P (*P < 0.05*). The ratios of MBC/MBN and C/N (65.4% and 13.5%) explained the most under No-Fert. treatment, respectively.

### 3.5 The relationship between soil ecological stoichiometry characteristics and forage yield

**Fig 5:**
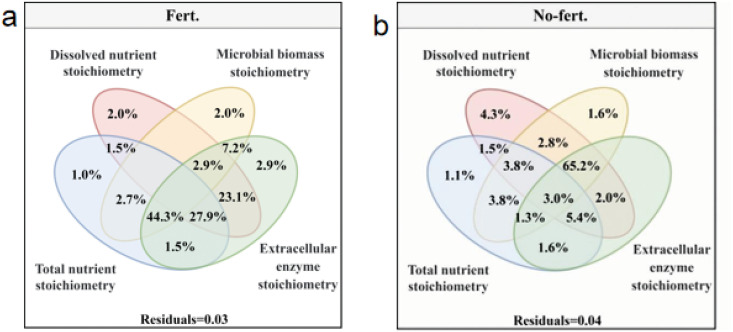
The effect of soil ecological stoichiometry on aboveground biomass indicators. Note: Variance partitioning analysis (VPA) was performed to determine the effect of soil ecological stoichiometry on forage yield indicators. Soil total nutrients stoichiometry (C/N, C/P and N/P); Soil dissolved nutrients stoichiometry (DC/DN, DC/DP and DN/DP); Soil microbial biomass stoichiometry (MBC/MBN, MBC/MBP and MBN/MBP) and soil extracellular enzyme stoichiometry (C/N-enzyme, C/P-enzyme and N/P-enzyme) under No-Fert. treatments. The significance level of the simple effect was P < 0.05* and P < 0.01**.

Under Fert. treatment, forage yield indicates were affected by the combined effect of soil microbial biomass stoichiometry, total nutrient stoichiometry and extracellular enzyme stoichiometry (44.3%), soil dissolved nutrient stoichiometry, total nutrient stoichiometry and extracellular enzyme stoichiometry (27.9%), soil dissolved nutrient stoichiometry and extracellular enzyme stoichiometry (23.1%) explains more than the other single and combined effect (Fig 5a). While forage yield indicates just affected by the combined effect of soil microbial biomass stoichiometry, soil dissolved nutrient stoichiometry and extracellular enzyme stoichiometry (65.2%) explains more than the other single and combined effect under No-Fert. treatment (Fig 5b)

## 4 Discussion

### 4.1 The effect of mixed-sowing on aboveground biomass

In present study, the aboveground biomass in the Ⅰ. + Ⅱ., Ⅰ. + Ⅲ. and Ⅰ. + Ⅱ. + Ⅲ. mixed combinations (except at gestation period) reached more than 70% of the total community yield (Table 2). This is mainly because the species of *El. N* is the dominant due to its large size and higher competitive ability [50,51]. As a result, it has access to more light resources in the mixed community and can quickly occupy a higher ecological position, dominating the mixed system [52,53]. The species of *E. nutans* and *P. albertii* subsp. poophagorum are the next most competitive with relatively weak growth. At gestation period, the contribution of *E. nutans* to forage yield in the Ⅰ. + Ⅱ. Ⅲ mixed combinations decreases to below 50%. In contrast, the contribution of *E. tangutorum* and *P. albertii* subsp. poophagorum to forage yield peaked. This is due to the fact that *E. nutans* grows faster early in the growing season, and its above-ground biomass dominants at the nodulation period [2]. In contrast, the species of *Po. A* grows slowly after re-greening, but reaches full bloom at the gestation stage.

Compared to the monoculture, the mixed communication of Ⅰ. + Ⅱ. + Ⅲ. had the highest forage yield of 2793.04 g·m^-2^, even for the unfertilized mixed combination (1363.09 g·m^-2^). The increase in plant diversity enhanced the overall resource utilization of the community that resulting in high yields [54,55]. Ecological niche differentiation in mixed seeding systems results in differences in the distribution of spatial, light and nutrient resources between species [56,57]. In present study, the ratio of RYT among all mixed combinations was greater than 1.0, except for the Fert. treatment of Ⅱ. + Ⅲ. (Fig 1). This indicates that the interspecific competition in mixed combinations is less than the intraspecific competition of the forage species [54] Different species will use ecological niche separation to achieve stable coexistence [58]. Discovering the mechanism of action between species diversity and productivity is a key issue in the study of diversity-productivity relationships [59,60]. The transgressive overyielding effect is evident in all mixed combinations, except for the Fert. treatment in the mixed combination of Ⅱ. + Ⅲ., supporting the hypothesis that interspecies competition in mixed systems is reduced compared to monoculture, thus leading to higher overall yield and greater ecological stability [61,62]. The results showed that there was a transgressive overyielding effect in all different treatments of mixed combinations, except for the Fert. treatment in the mixed combination of Ⅱ. + Ⅲ., and both OY_1_ and OY_2_ were higher in he mixed combination of Ⅰ. + Ⅱ. + Ⅲ. than in the other mixes (Fig 2).

### 4.2 The effect of fertilizer application on aboveground biomass and con-existence of species in mixed combinations

Fertilizer application as a management practice is a key factor in regulating forage yield in grassland ecosystems [38,63]. Compared to No-Fert. treatment, an increasing in aboveground biomass at all stages of the growing season in mixed combinations under fertilizer conditions (Table 2). Forage productivity of tall plant *E. nutans*, medium height *E. tangutorum* and low *P. albertii* subsp. poophagorum all responded positively to the fertilizer treatment. This positive response of aboveground biomass to fertilizer treatment reduces the overlap of species’ ecological niches, reduces environmental competition between species and promotes species coexistence [64,65]. Although *E. nutans* and *E. tangutorum* are conspecific and have similar resource requirements, intraspecific competition is greater than interspecific competition for the dominant species *E. nutans* [2,3]. This means that conspecific individuals are more similar in resource requirements than heterospecific individuals [66,67]. This is the reason why in mixed combination Ⅰ. + Ⅱ., species *E. nutans* and *E. tangutorum* are still able to co-exist and obtain transgressive overyielding. The positive effect of below-ground biomass of grasses and fertilizer application, increasing soil nutrients and significantly increasing community productivity through the return of organic matter by root secretions and below-ground biomass [68]. In addition, competition between grassland species is asymmetrical and the competitiveness derived from this can change depending on the age class of the grassland.

Under fertilizer conditions, the increase in forage yield of *P. albertii* subsp. poophagorum was less than that of *E. nutans* and *E. tangutorum* compared to the above-ground biomass, the competitive advantage of the *E. nutans* root system became weaker, and the species of *P. *albertii* subsp. poophagorum* was more competitive with a stronger growth potential. Different species respond asynchronously to environmental perturbations and there are differences in species’ responses following fluctuating environments [69,70]. The positive effect of fertilizer application was more pronounced for the *E. nutans* root system, while the competitive advantage of *P. albertii* subsp. poophagorum increased, suggesting a shift in competitive dynamics. This aligns with our hypothesis that species respond differently to fertilizer application, with the overall increase in biomass and transgressive overyielding primarily driven by fertilization (Table 1 and Fig 2). Therefore, the effect of transgressive overyielding in mixed combinations in this study was mainly due to fertilizer application. For artificial grasslands established to cultivate forage, appropriate amounts of supplementary fertilizer can compensate for nutrient loss from the soil over time and promote C cycling [71], as well as improve grassland productivity. The selection of high-yielding, low-carbon native grass species to complement planting and management practices is crucial for the sustainable development of the Tibetan Plateau.

### 4.3 The response strategies of soil ecological stoichiometry characteristics to mixed combinations and fertilizer application

The diversity of nutrient use strategies among species plays a crucial role in regulating ecosystem structure, function, stability, and diversity [72] Altering mixed species combinations and adding nitrogen (N) fertilizers can influence soil nutrient status, thereby promoting forage growth and increasing yields [73]. Central to ecological stoichiometry theory is the concept of biochemical homeostasis [74]. Soil microorganisms are pivotal in linking various ecosystem functions and services within the framework of ecological stoichiometry [75]. Their resource dependency represents a robust adaptation strategy for maintaining homeostasis [76]. In this study, soil ecological stoichiometry responded differently to aboveground biomass and indicators of transgressive overyielding under varying mixed species combinations and fertilizer treatments in artificial grasslands. This indicates that stoichiometric characteristics readily utilized by microorganisms play a regulatory role in forage yield and transgressive overyielding under unfertilized conditions. Levels of DC, DN, and MBC/MBN in the soil determine species coexistence and yield in mixed species combinations. The positive correlation between forage yield, transgressive overyielding coefficients, and microbial biomass stoichiometry (MBC/MBN) under non-fertilized conditions supports the hypothesis that microbial stoichiometry plays a crucial regulatory role in mixed communities under natural nutrient conditions (Fig 3 and S1 Table) [77]. Under strict homeostasis, changes in resource stoichiometry have minimal impact on organism stoichiometry. However, despite maintaining relative homeostasis, soil microbial biomass exhibits sensitivity to C: N ratios [74].

In contrast, aboveground biomass and transgressive overyielding coefficients of grassland were only significantly positively correlated with MBC/MBN in the Fert. treatments and were constrained by N/P, C/P and C/P-enzyme (Fig 4 and S2 Table). That means that forage yield and over-yield coefficients under fertilizer treatments are limited by P. Although an important mechanism for forage to maintain their survival in a severely P-limited environment through high nutrient recovery and utilization of their own P at a constant rate [25]. Nitrogen and phosphorus are the main nutrients that limit plant growth and can characteristics the nutrient limitation. Therefore, they are widely used to analysis N and P limitation in ecosystems, communities and plant populations [78]. The ratio of N/P can determine community structure and function [79]. When N/P < 14, plant growth is limited by N; when N/P > 16, plant growth is limited by P; when 14 < N/P < 16, plant growth is limited by both N and P [80]. The results showed that under the No-Fert. treatment I. + III., II. + III. and Ⅰ. + II. + III. The soil DN/DP of the mixed combinations were all greater than 16, forage growth was limited by P. The fertilization process disrupts the microbial homeostasis of the soil native system, and each of the P-related stoichiometric traits corresponds to community coexistence and yield. The main mechanism of species coexistence in plant communities is the slightly different nutrient use of N and P by different species, i.e. the differentiation of the nutritional ecological niche of the community [81]. It is a common fact that N deficiency in soils limits the productivity of grasslands, and compensating for degraded grasslands through fertilization measures is the key to increasing forage yield. The right amount of N fertilizer application can improve the yield and quality of forage and increase the accumulation of N in forage [73,82]. Forage yield was significantly greater in the Fert. treatment than in the No-Fert. treatment (Table 1). The addition of N addresses to a certain extent the problem of insufficient soil nitrogen content, thus promoting plant growth, increasing plant biomass and the input of organic carbon to the vegetation and effectively improving ecosystem productivity and forage quality [83,84].

The response strategies of soil ecological stoichiometry under fertilizer and unfertilized treatments are quite different [85,86]. Under the No-Fert. treatment, soil microorganisms initiated strong adaptive strategies to maintain their homeostasis. Thus, under the resource dependence of soil dissolved nutrient stoichiometry, microbial-related soil microbial biomass stoichiometry and extracellular enzyme stoichiometry characteristics that contributed 65.2% of the explanation for forage yield. In contrast, under the fertilizer treatment, the first response of soil dissolved nutrient stoichiometry leads to a change in total nutrient stoichiometry, causing soil microorganisms to initiate extracellular enzymes to coordinate the change in resources. In the systems of plant-microbial-soil, microbially mediated soil C, N and P cycling is always dynamic [87], which may result in an imbalance between substrate supply and microbial demand [31]. Microorganisms need to adapt to metabolic limitations caused by inadequate substrate resource availability by adjusting their own elemental utilization efficiency or by producing specific extracellular enzymes to mobilize resources in order to maintain normal life activities [88,89]. Thus, soil microbial biomass stoichiometry, total nutrient stoichiometry and extracellular enzyme stoichiometry (44.3%), soil dissolved nutrient stoichiometry, total nutrient stoichiometry and extracellular enzyme stoichiometry (27.9%), soil dissolved nutrient stoichiometry and extracellular enzyme stoichiometry combined to affect forage yield under the fertilizer treatments.

## 5 Conclusion

The aboveground biomass peaked at the end of the growing season, with the highest biomass found in the *E. nutans + E. tangutorum + P. albertii* subsp. poophagorum mixed sowing combination, supporting the hypothesis that plant diversity enhances productivity through transgressive overyielding. Fertilizer application and mixed sowing significantly increased community productivity, confirming that both factors positively impact forage yield and ecosystem functioning.

Soil ecological stoichiometry responded differently to fertilized and unfertilized treatments. Under the No-Fert. treatment, soil microorganisms regulated soil C, N, and P via dissolved nutrient stoichiometry, influencing aboveground biomass. In contrast, fertilization altered extracellular enzyme stoichiometry, balancing substrate supply and demand. These findings highlight the role of species diversity and nutrient management in grassland restoration, with the transgressive overyielding effect demonstrating the benefits of mixed sowing for improving productivity and stability

## Supporting information

S1 Table Commission number, abbreviation and corresponding substrate of soil extracellular enzymes. (DOCX)

S1 Fig Soil total nutrients stoichiometry characteristics. (DOCX)

S2 Fig Soil dissolved nutrients stoichiometry characteristics. (DOCX)

S3 Fig Soil microbial biomass stoichiometry characteristics. (DOCX)

S4 Fig Soil extracellular enzyme stoichiometry characteristics. (DOCX)

S1 Dataset Data on aboveground biomass, soil total nutrient, soil dissolved nutrient and soil extracellular enzymes under different grass pasture in the northern Tibetan Plateau. (XLSX)
