# Supplementary material for: Short-time response of soil ecological stoichiometry on aboveground biomass under fertilizer application of mixed grass pasture in the Northern Tibetan Plateau
Source: PLoS One. 2025 Jul 21;20(7):e0326265. doi: 10.1371/journal.pone.0326265 (PMC12279096; doi:10.1371/journal.pone.0326265)
Supplement: S1 Table — (DOCX) [file pone.0326265.s001.docx]

**Table S1. Commission number, abbreviation and corresponding substrate of soil extracellular enzymes.**

| EEAs | Abbreviation | ECN | Corresponding substrate |
| --- | --- | --- | --- |
| β-1,4-glucosidase | BG | 3.2.1.21 | 4-MUB-β-D-glucoside |
| β-D-cellobiohydrolase | CBH | 3.2.1.91 | 4-MUB-β-D-cellobioside |
| β-N-acetylglucosaminidase | NAG | 3.2.1.14 | 4-MUB-N-acetyl-β-D-glucosaminide |
| Leucine-aminopeptidase | LAP | 3.4.11.1 | L-leucine-7-amido-4-methylcoumarin hydrochloride |
| Alkaline phosphatase | AP | 3.1.3.1 | 4-MUB-phosphate |
