# Supplementary figures and images for: Short-time response of soil ecological stoichiometry on aboveground biomass under fertilizer application of mixed grass pasture in the Northern Tibetan Plateau
Source: PLoS One. 2025 Jul 21;20(7):e0326265. doi: 10.1371/journal.pone.0326265 (PMC12279096; doi:10.1371/journal.pone.0326265)

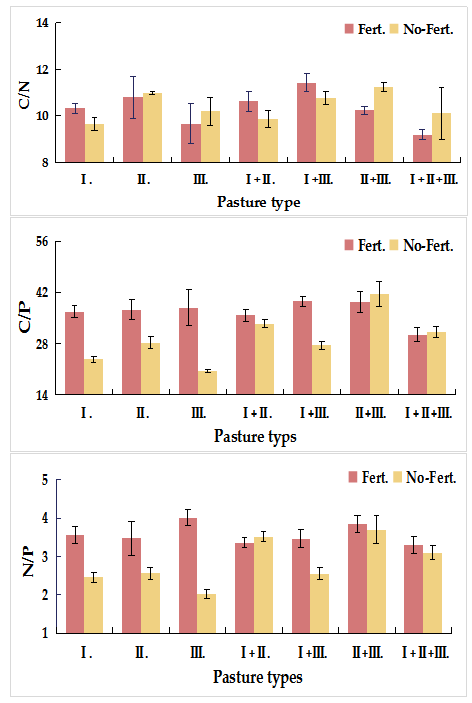


**Fig. S1 Soil total nutrients stoichiometry characteristics.**

Supplement: S1 Fig — (DOCX) [file pone.0326265.s002.docx]

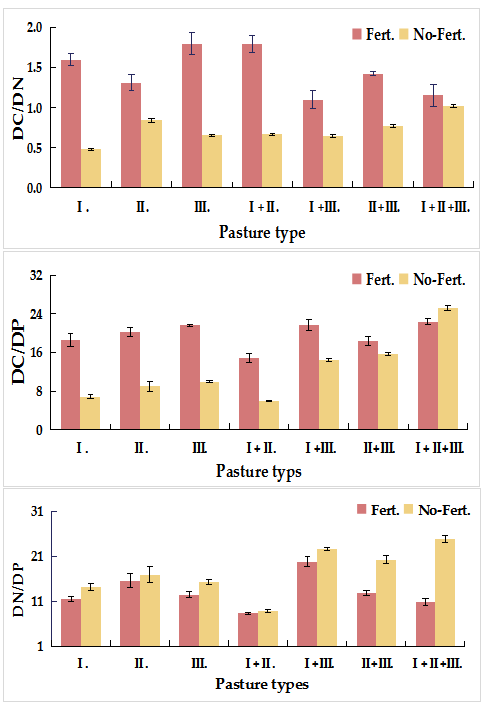


**Fig. S2** **Soil dissolved nutrients stoichiometry characteristics.**

Supplement: S2 Fig — (DOCX) [file pone.0326265.s003.docx]

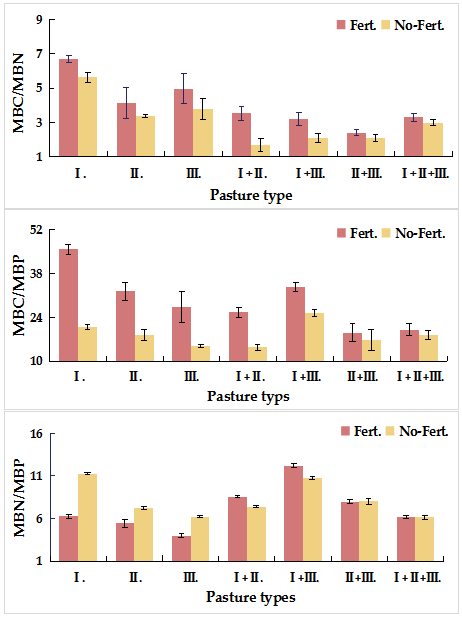


**Fig. S3 Soil** **microbial biomass stoichiometry characteristics.**

Supplement: S3 Fig — (DOCX) [file pone.0326265.s004.docx]

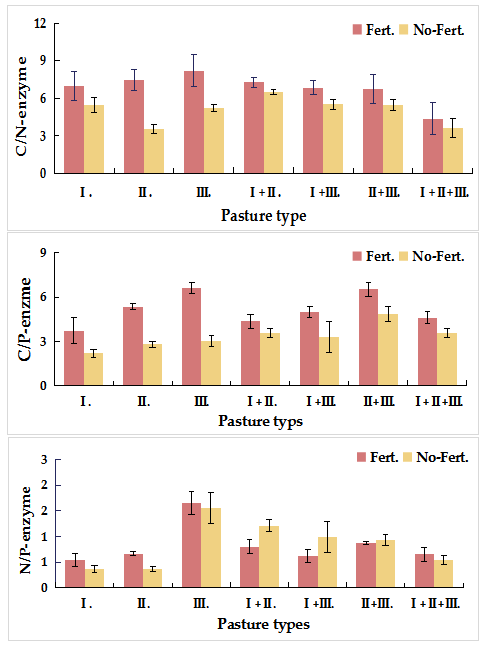


**Fig. S4 Soil** **extracellular enzyme stoichiometry characteristics.**

Supplement: S4 Fig — (DOCX) [file pone.0326265.s005.docx]
